# Can pill placebo augment cognitive-behavior therapy for panic disorder?

**DOI:** 10.1186/1471-244X-7-73

**Published:** 2007-12-20

**Authors:** Toshi A Furukawa, Norio Watanabe, Ichiro M Omori, Rachel Churchill

**Affiliations:** 1 Department of Psychiatry and Cognitive-Behavioral Medicine, Nagoya City University Graduate School of Medical Sciences, Mizuho-cho, Mizuho-ku, Nagoya 467-8601, Japan; 2 Section of Evidence-Based Mental Health, Health Services Research Department, Institute of Psychiatry, King's College London, University of London, UK

## Abstract

**Background:**

In a number of drug and psychotherapy comparative trials, psychotherapy-placebo combination has been assumed to represent psychotherapy. Whether psychotherapy plus pill placebo is the same as psychotherapy alone is an empirical question which however has to date never been examined systematically.

**Methods:**

We conducted a systematic review and meta-analysis of randomised controlled trials (RCTs) that directly compared cognitive-behavior therapy (CBT) alone against CBT plus pill placebo in the treatment of panic disorder.

**Results:**

Extensive literature search was able to identify three relevant RCTs. At the end of the acute phase treatment, patients who received CBT plus placebo had 26% (95%CI: 2 to 55%) increased chances of responding than those who received CBT alone. At follow-up the difference was no longer statistically significant (22%, 95%CI: -10% to 64%).

**Conclusion:**

The act of taking a pill placebo may enhance the placebo effect already contained in the effective psychotherapeutic intervention during the acute phase treatment. Theoretically this is an argument against the recently claimed null hypothesis of placebo effect in general and clinically it may point to some further room for enhancing the psychotherapeutic approach for panic disorder.

## Background

The observed treatment effect, i.e. the change from baseline till endpoint, is traditionally thought to be due to four factors: regression towards the mean, natural course of disease, placebo effect i.e. non-specific effects of the therapist and the setting in which therapy takes place, and specific effects of physical or psychological intervention on the target condition [1].

When a specific therapy for a certain disorder exists, we therefore assume that it realizes all these four components through its administration. Cognitive-behavior therapy (CBT) for panic disorder is one such instance, because it has demonstrated its superiority in the rigorously internally calibrated, drug-sensitive [2] group of patients vis-à-vis the pill placebo arm [3] and also against a non-specific psychological intervention [4].

Whether adding pill-placebo to specific psychotherapy enhances its effectiveness is an empirically and theoretically interesting question [5]. Some may assume that a specifically effective treatment has already realized all the above-mentioned four components of treatment effect and therefore addition of a pill placebo cannot enhance its effect. On the other hand, some may suspect that addition of pill placebo may subtract from psychological treatment because it may undermine the active commitment of the patient to follow psychological interventions. Or, some may argue that psychotherapy placebo and pharmacotherapy placebo work through different psychological mechanisms and can therefore be additive or synergistic.

While conducting a comprehensive systematic review of randomized controlled trials (RCT) of the combined psychological and drug treatment for panic disorder [6,7], we had a unique opportunity to compare the psychotherapy alone versus the psychotherapy plus pill placebo arms and would like to repot the results.

## Methods

### Identification of trials

In order to identify all randomized controlled trials that compared psychotherapy against psychotherapy plus placebo, we looked for all trials that examined the combination of psychotherapy and pharmacotherapy by antidepressant or benzodiazepines, two types of drugs known to be effective for panic disorder.

Both individual and group formats of the following psychological treatments were included: behavior therapy involving some kind of exposure, cognitive therapy which uses some kind of cognitive restructuring, cognitive-behavior therapy involving elements of both cognitive and behavioral therapies, and other psychological interventions. All commonly prescribed antidepressants and benzodiazepines were eligible.

We electronically searched the Cochrane Collaboration Depression, Anxiety and Neurosis Controlled Trials Register (CCDANCTR) with keywords *antidepressant *OR *benzodiazepines *and *panic *in April 2003 and October 2005. The CCDANCTR is a study-based register of randomized trials incorporating results of group searches of MEDLINE (1966-), EMBASE (1980-), CINAHL (1982-), PsycINFO (1974-), PSYNDEX (1977-) and LILACS (1982–1999) and handsearches of major psychiatric and medical journals. Two complementary searches for additional relevant trials were conducted with the Cochrane Central Register of Controlled Trials (CENTRAL) and with MEDLINE. No language restriction was imposed.

Two reviewers examined titles and abstracts of studies identified by the electronic search strategies and then checked full articles for eligibility. To identify further trials, references of these selected studies and of other review papers were also checked, representative studies were subjected to SciSearch, and experts were contacted.

### Quality assessment and data extraction

Two independent reviewers (NW and TAF) assessed the methodological quality of the selected studies. The criteria for quality assessment were based on recommendations in the Cochrane Handbook for Systematic Reviews of Interventions [8], which focused on the quality of allocation concealment. We also rated whether at least one outcome measure was assessed by an independent assessor blind to treatment allocation.

Two reviewers independently extracted data from the original reports using data extraction forms. For studies where exact numbers of responders were not presented but only their means and standard deviations (SDs) of the global severity measure, we imputed response rates by using a validated imputation method [9] in order to conduct intention-to-treat (ITT) analyses as described below. Any disagreement was resolved by consensus between the first three reviewers.

### Data synthesis

Our primary outcome was "response," i.e. substantial improvement from baseline as defined by the original investigators. Examples would be "very much or much improved" according to the Clinical Global Impression Change Scale [10], and more than 40% reduction in the Panic Disorder Severity Scale [11] score or in panic frequency. The total number of dropouts for any reason was regarded as a proxy measure of treatment acceptability. Adverse effects were evaluated by looking at the number of dropouts due to adverse effects.

For dichotomous outcomes, ITT analysis was adopted. When dropouts were excluded from any assessment in the primary studies (for example, those who never returned for assessment after randomization), they were considered non-responders. Relative risks (RR) and their 95% confidence intervals were calculated using random effects model rather than a fixed effects model because of its generalizability [12].

Heterogeneity, which refers to variability among studies in a systematic review and generally derives from clinical, methodological or statistical diversity [8], was assessed by Chi-squared statistics and I-squared statistics [13].

## Results

### Description of studies

The electronic search identified 274 studies from CCDANCTR, 231 from CENTRAL and 35 from MEDLINE. Browsing their titles and abstracts, 195 articles were identified by either of the two independent reviewers as possible candidates and their full copies were obtained. With further reference search, SciSearch and personal contacts, we identified 25 studies that examined the combination of psychotherapy plus pharmacotherapy for panic disorder. Of these, three studies included psychotherapy plus placebo as well as psychotherapy alone arms: Barlow *et al.*[3], de Beurs *et al.*[14], and Sharp *et al.*[15]. Table 1 gives details of the participants' characteristics, the interventions, and the definition of response in these three trials. All three trials examined behavioral and cognitive therapies and in the following we summatively refer to them as CBT.

**Table 1:** Characteristics of the included studies

Study	Participants	Interventions	Outcomes
Barlow et al (2000)	DIAGNOSIS: DSM-III-R panic disorder with mild to no agoraphobia AGE: mean = 34.1 to 37.8 years SEX: 62% women	12 weeks of: 1. imipramine (mean = 214 to 239 mg/d by week 12) + CBT (11 sessions during 3 months) 2. imipramine alone 3. CBT alone 4. CBT + placebo 5. placebo alone	RESPONSE: > = 40% reduction on PDSS
De Beurs et al (1995)	DIAGNOSIS: DSM-III-R panic disorder with moderate to severe agoraphobia AGE: mean = 38.8 SEX: 75% women	12 weeks of: 1. fluvoxamine (100–150 mg/day) + exposure (12 weekly sessions) 2. exposure alone 3. placebo + exposure 4. psychological panic management plus exposure	RESPONSE: > = 40% reduction in panic frequency [imputed from its mean & SD]
Sharp et al (1996)	DIAGNOSIS: DSM-III-R panic disorder with or without agoraphobia (% agoraphobia unclear; however, the average score for FQ-Ag was around 15, indicating most had at least some agoraphobia) AGE: mean = 33.2 to 38.8 SEX: 78% women	12 weeks of: 1. fluvoxamine (100–150 mg/day) + CBT (12 sessions) 2. fluvoxamine alone 3. CBT alone 4. CBT + placebo 5. placebo alone	RESPONSE: "Very much" or "Much improved" on CGI Change

PDSS: Panic Disorder Severity Scale, CGI: Clinical Global Impression

### Acute phase treatment

**Figure 1:**
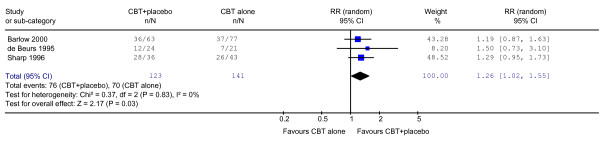
Response on CBT + placebo vs CBT alone in the acute phase treatment.

At the end of the acute phase treatment which lasted 12 weeks in all the three trials, patients who received CBT plus placebo were 1.26 (95%CI: 1.02 to 1.55) times more likely to show response than those who received CBT alone (Figure 1). There was no statistical heterogeneity among the three trials.

Treatment acceptability, as measured by the total number of dropouts for any reasons, did not differ between the two groups (RR = 0.83, 0.40 to 1.72). There were no dropouts due to side effects in psychotherapy alone or psychotherapy plus placebo arms of the three trials.

### Follow-up after trial termination

**Figure 2:**
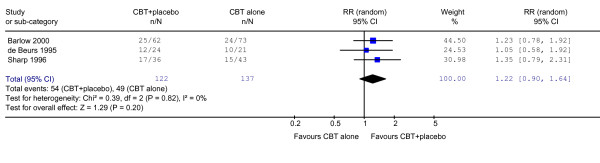
Response on CBT + placebo vs CBT alone at 6–24 months after acute phase treatment.

At follow-up 6 to 24 months after termination of the trials, psychotherapy plus placebo was no longer statistically significantly superior to psychotherapy alone (RR = 1.22, 0.90 to 1.64). The I-squared statistic was 0%, indicating no heterogeneity in the pooled results (Figure 2).

## Discussion

The present report represents the first systematic empirical examination of the effect of adding pill placebo to psychotherapy. It found that, for panic disorder with or without agoraphobia, pill placebo may enhance CBT during the acute phase treatment. There was no difference in treatment acceptability between these two treatment arms. The difference in effectiveness, however, seemed to wane in 6–24 months after end of the acute phase treatment.

Twenty-five years ago, Hollon and DeRubeis argued that placebo-psychotherapy combination cannot represent psychotherapy in drug-psychotherapy comparative trials but could not determine from the literature review then whether the former would overestimate or underestimate the latter [5]. The current study has assembled newer data and suggests that placebo-psychotherapy combination overestimates the latter and that therefore Hollon and DeRubeis's concerns proved justified.

The fact that pill placebo can add to psychotherapy is intriguing. Theoretical implications of the present findings may be two. Firstly, in contrast to some old claims [16], taking a pill placebo did not undermine the effectiveness of psychological intervention. Secondly, much to the contrary, pill placebo enhanced the effectiveness of CBT. The act of taking an inactive drug can enhance the placebo effect already contained in the effective psychotherapeutic intervention. This is a strong argument against the recently claimed null hypothesis of placebo effect [17].

The observed pill placebo effect over and above the proven psychotherapy may be explainable from both of the main theoretical models of the placebo effect, namely the classical conditioning [18] and expectancy theory [19] In the life of a modern man the beneficial experience of a pill is almost inescapable and this may have contributed to the placebo effect through pill taking in addition to the same through talk therapy. In other words, the pill has become a conditioned stimulus, eliciting a conditioned response which is placebo effect. Or alternatively, according to the expectancy theory, patients expect more from pill and talk therapy so that they respond more to pill and talk therapy. The fact that the benefit of the pill placebo appeared to wane after the treatment termination is compatible with both of these theoretical models. In fact, although they are often presented as competing perspectives, the two theories are not necessarily incompatible with each other [20].

Pragmatic implications of the present findings may not be as straightforward. We have formerly established that antidepressant drug plus CBT was more effective than placebo plus CBT at the end of the acute phase treatment [6]. It appears that psychotherapy plus pill placebo would come in-between the two, but in clinical practice prescribing a pill placebo will be very difficult if not unethical. It is important to note, however, that there is some further room to enhance placebo effect when administering CBT to panic disorder.

There are some possible weaknesses to the present study. In the first place, despite our intensive and extensive literature search, we were able to identify only three trials that made a head-to-head comparison between psychotherapy plus placebo against psychotherapy alone. The present findings therefore may lack statistical power and should be regarded exploratory and preliminary. Secondly, in two of the three included studies, the primary outcomes were rated by raters who were not blind to the psychotherapy status but who were blind only to the drug status (active drug vs placebo). However, we must note that the absolute degree of heterogeneity, as expressed by I-squared values and as visually observable in forest plots (Figures 1 and 2), was close to zero among the one blind and two non-blind studies. The open nature of the assessment can therefore not explain away the observed difference. Thirdly, the response rate had to be imputed in one of the three studies [14] based on the panic frequency, which likely not reflect the whole panic disorder psychopathology. Deleting this study, however, did not affect the outcomes (RR = 1.24, 95%CI: 1.00 to 1.54 at the end of acute phase treatment, and RR = 1.28, 95%CI: 0.91 to 1.80 at 6–24 months after treatment discontinuation). We were also unable to conduct detailed meta-analyses of continuous variables presented in the identified studies because each rated some different aspects of panic disorder. For example, one RCT reported frequency of panic attacks, two reported on phobic avoidance, two reported on general anxiety, one reported depression and one reported social dysfunction on various continuous scales. The field would certainly benefit from wider-spread use of a more common metric of overall panic disorder severity, such as the Panic Disorder Severity Scale [11]. Lastly, our results concern panic disorder only and generalizability beyond this disorder is not guaranteed. More systematic reviews on this topic in other disorders are warranted.

## Conclusion

The act of taking a pill placebo may enhance the placebo effect already contained in the effective psychotherapeutic intervention during the acute phase treatment. There may be some further room for enhancing the psychotherapeutic approach for panic disorder.

## Competing interests

The author(s) declare that they have no competing interests.

## Authors' contributions

TAF conceived of the study, and drafted the protocol. All the authors discussed and amended the study protocol. TAF, NW and IMO did the study selection, data extraction and data entry. TAF analyzed the data and wrote the first draft of the manuscript. All the authors discussed and amended the manuscript, and approved the final manuscript.

## Pre-publication history

The pre-publication history for this paper can be accessed here:


